# Overview of prevention and treatment approaches: a summary from the European association for research on obesity in childhood “Prevention of Obesity” symposium

**DOI:** 10.3389/fped.2025.1607237

**Published:** 2025-09-02

**Authors:** Paula Moliterno, Kurt Widhalm

**Affiliations:** ^1^ Austrian Academic Institute for Clinical Nutrition, Vienna, Austria; ^2^ Department of Pediatrics, Medical University of Vienna, Vienna, Austria

**Keywords:** obesity, childhood, Europe, prevention, symposium

## Abstract

With childhood obesity being one of the most common pediatric chronic diseases, years of extensive prevention experiences still show shortcomings. To gather a discussion over first-hand obesity prevention interventions, the European Association for Research on Obesity in Childhood and the Austrian Academic Institute for Nutritional Medicine organized an international symposium on “Prevention of Obesity” in October 2023 (Vienna, Austria). . This report aimed to summarize what researchers from Austria, Denmark, France, Germany, Italy, Portugal, Sweden, and Norway presented and discussed regarding different childhood obesity prevention (and treatment) models implemented in their countries. Using the Ecological Systems Theory framework, we mapped strategies across individual, interpersonal, organizational, community, and policy levels. Family-based interventions were considered best practices; education and counseling on parenting in the context of pediatric obesity is key, as well as using incentives, reminders, and feedback support. Financial support through sufficient personnel and materials budgets, interventions implementation, ongoing result analysis, and program sustainability was considered crucial for long-term success in preventing obesity. While most strategies focused on individual, interpersonal, and organizational levels, fewer directly addressed the community or policy levels, highlighting opportunities for more integrated, system-wide approaches.

## Introduction

Childhood obesity is now considered one of the most common pediatric chronic diseases, with reports projecting that its global prevalence will increase by 100% between 2020 and 2035 (1). This public health problem requires collective efforts and a whole system approach. For many years, evidence-based experiences on prevention strategies have been developed, aiming at finding which lifestyle, behavioral, environmental, and policy intervention components approaches are most successful, and what is necessary to prevent excessive weight gain from the prenatal period and continuing through childhood and adolescence. On October 21, 2023, the European Association for Research on Obesity in Childhood (EAROC) and the Austrian Academic Institute for Nutritional Medicine, led by Prof. Dr. Kurt Widhalm, organized an international symposium on “Prevention of Obesity.” Renowned European researchers presented first-hand prevention and treatment interventions targeting children, families, and communities. All presentations at the event—regardless of the presenters' association membership—were included for this symposium report. The selection criterion was comprehensive inclusion, ensuring that every prevention and management strategy showcased at the symposium in October 2023 is represented in this publication. The symposium's presentations were organized using Ecological Systems Theory (EST) (2) as a guiding framework to summarize strategies addressing childhood obesity, including both preventive approaches aimed at reducing risk factors before excess weight gain occurs as well as interventions targeting children and adolescents already living with overweight or obesity (Appendix 1 Table 1). Characteristics that place children at a higher risk of developing obesity were included, considering children's ecological niche (family and school) at individual and interpersonal levels and the influence of organizational and community characteristics and public policy factors (2, 3). This integrated approach reflects the spectrum of efforts discussed at the symposium, from primary prevention to treatment and management strategies.

## The symposium

The symposium opened by acknowledging that prevention is an excellent and reputable investment as it generates an economic return: every dollar spent on preventing obesity generates up to six dollars of economic return (4). Childhood obesity incurs additional healthcare costs compared to children of normal weight (4).

Moreover, the long-term registry APV (Adipositas-Patienten-Verlaufsdokumentation) initiative has shown the difficulties that children experience in weight reduction, as 36.3% of children experienced no loss after 2 years of an outpatient lifestyle intervention (5). In this sense, family doctors' and pediatricians' roles as first-hand health professionals were highlighted as they should help identify and address modifiable early-life risk factors for obesity in childhood.

**Figure 1:**
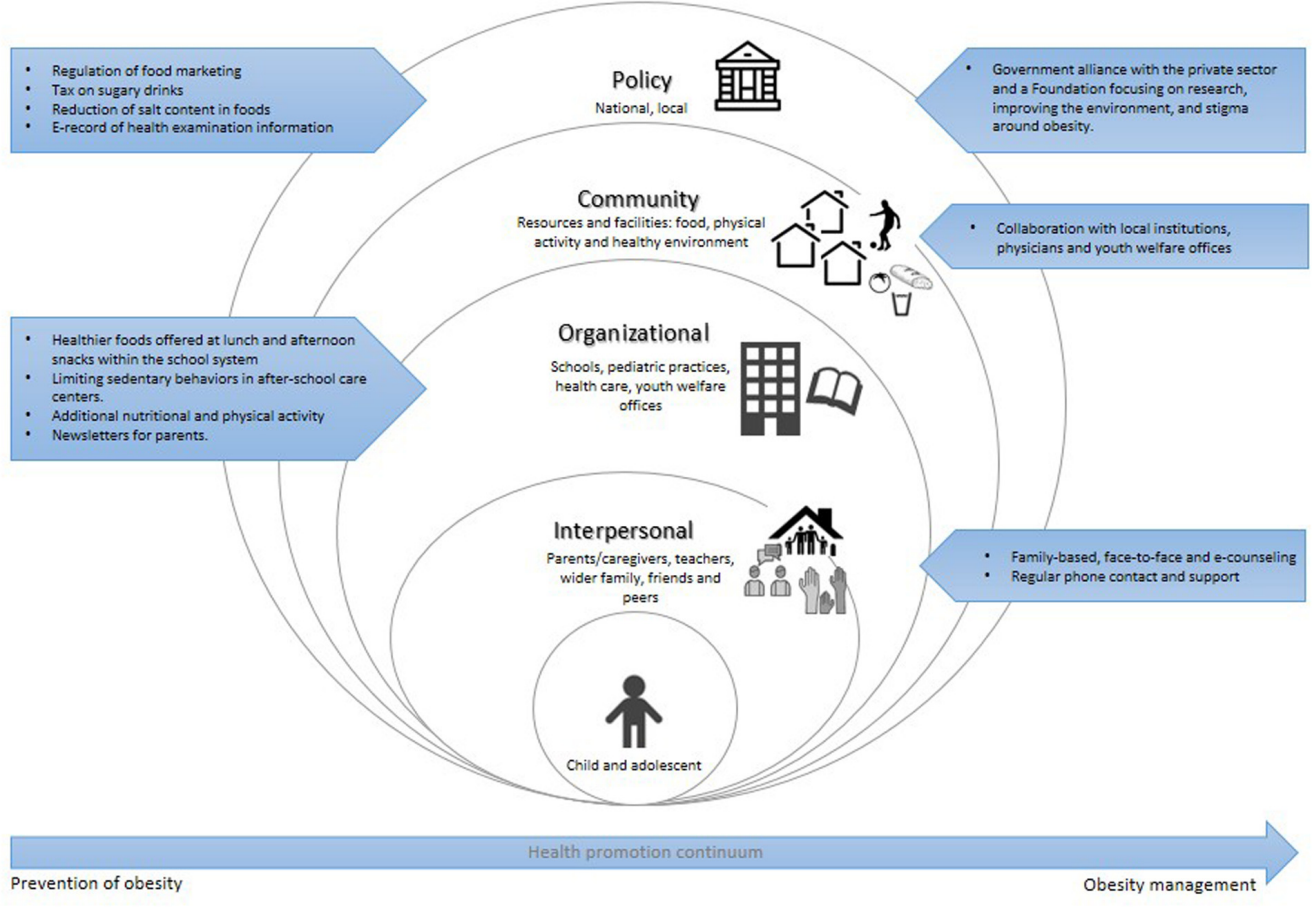
Prevention and management of childhood obesity within the ecological systems theory framework. The ecological systems theory model (2) comprises different levels, from the individual level to public policy. Interventions targeting the individual and interpersonal domains can be described as downstream interventions, and interventions within policy can be described as the highest level of upstream interventions. Within each level, arrows are shown, corresponding to prevention (left) and management (right) of obesity strategies presented at the symposium.

The World Health Organization (WHO) has proposed advocating for structural condition-oriented obesity prevention, recommending adopting of a whole-system strategy when designing and implementing preventive measures (6). The latter implies moving away from isolated, single-level interventions and promoting the integration of health-promoting policies, community resources, educational systems, and environmental modifications critical in obesity risk and prevention, to create supportive conditions for healthy choices. By doing this, feasible and effective strategies can be achieved. In alignment with these recommendations, the symposium presentations were organized according to the Ecological Systems Theory (EST) framework (Figure 1). Although conceptually distinct, prevention and treatment of childhood obesity are often intertwined in practice. Moreover, management of childhood obesity could be regarded as the prevention of adult obesity (7). Public health and health care services' response to obesity must form a continuum and ideally integrate the obesity prevention model with managing excessive weight (6, 8). Stopping the rise in obesity demands an integrated multisectoral approach that addresses prevention and treatment, reflecting the continuum of strategies needed to combat childhood obesity in ecological contexts. This approach facilitated a comprehensive analysis strategies implemented across various European contexts, with particular attention to interventions at the individual, interpersonal, organizational, community, and policy levels. We aim to organize the symposium's presentations through this structure to help understand and identify opportunities for integrated, system-wide approaches that help to understand and simultaneously address prevention and treatment of child and adolescent obesity.

### Individual and interpersonal level

The individual level considers those interventions targeting children's knowledge, attitudes, skills, and behaviors, while the interpersonal level emphasizes the influence of children's social networks and support systems on their behavior (2). The WHO Commission on Ending Childhood Obesity has posed that targeting individual behaviors alone may be associated with failure in obesity prevention (9). Regarding childhood obesity, it is clear that the interpersonal level plays a fundamental role as decisions on eating and moving habits and lifestyle choices begin at home and are strongly related to their parents and wider family influence. Moreover, children are immersed in settings that indirectly foster behaviors, such as the school environment (6). As children spend several hours of the day at school, these organizational settings offer a magnificent opportunity to promote individual healthy skills and knowledge about nutritious food and the importance of a healthy and active lifestyle. However, they also influence, at an interpersonal level, peers and family to consolidate sustainable obesity prevention measures. Presentations targeting the school setting will be discussed at the organizational level.

Several family-based presentations targeting the family environment were presented; however, approaches focused on healthy weight achievement strategies to address those already affected by overweight and obesity. The STARKIDS program in Germany (10) was a cluster-randomized controlled trial implemented for children and adolescents with overweight/obesity between 3 and 17 years. It focused on the context of their whole family, as a new approach combining face-to-face counseling in low-barrier environments such as the pediatric practice in combination with personalized e-health interventions. This project's added value is the family support and combined approach, which also focuses on quality of life. The design study included two parallel arms: the intervention group that received the STARKIDS program and the control group that received standard treatment in pediatric practices, which were randomly allocated to either the intervention or the control condition. The intervention consisted of two steps. The first step consisted of face-to-face training in the pediatric practice and an e-health online platform. Every 3 months, families underwent a 90-min-long face-to-face training, each covering one module (“STARKIDS Start,” “Eating and Drinking,” “Activity and media,” “Family life,” “STARKIDS Keep on,” and the optional “STARKIDS Joker”) in their pediatric practice, thus continuing for 1 year from first to last face-to-face training. A medical assistant taught all training using the spirit of motivational interviewing. Step 1 of the STAR KIDS program lasted 1 year, after which, by pre-defined criteria of BMI reduction, a decision on whether the participant should use step 2 is applied. If BMI was not reduced as expected, they received counseling (1–2 counseling sessions in a 6-month, by participating in public health services) about further programs and opportunities nearby, depending on the area families are still struggling with. As a plus, all families could use the STARKIDS online platform for the next six months, after which the program would conclude. On the contrary, families in the control group received face-to-face, one-time structured counseling (approximately 30 min) provided by medical assistants concerning healthy weight development, diet, physical activity, media consumption, sleeping habits, and stress. However, the latter was limited to providing guideline-based recommendations and did not provide further tools. Families received a flyer summarizing the guidelines for healthy weight development at the end of their one-time structured counseling. Outcomes were measured in three measurement times-points: at baseline/inclusion in the study, baseline + 12 months, and baseline + 18 months. Primary outcomes were weight development measured by BMI standard deviation scores and subjective positive mean change in children's and adolescents' quality of life from baseline to follow-up. Additional outcomes related to the modules covered were measured through questionnaires. With the proof of concept, STARKIDS, which concluded in December 2024, finally included 611 participants and provides the potential to be implemented as a standard care tool for the prevention and intervention of childhood/adolescent obesity in the German health system (10). Preliminary data regarding participants' experience in the project has been published (11); however, no full outcome data has been published.

The ProxOb project in France, another family-based initiative, also targeted children and families with obesity and aimed to test a “real-life” approach that included the whole family in its natural environment and routine (12). The initiative allowed the participation of families with at least one child under 18 living with overweight or obesity or a child under 6 (before adiposity rebound) with both parents living with obesity. Additional requisites were being part of a family with a precarious social situation, living in an area with reduced or no healthcare access, or having experienced unsuccessful previous obesity interventions. Families must also be available for project evaluations and welcome practitioners into their homes during the intervention. The project, which aligned with the objectives of the French National Obesity Plan and endorsed as innovative by the French Minister of Health, provided families with a 6-month multidisciplinary, home-based, and family-centered intervention followed by an 18-month maintenance phase, with regular phone contact and support (12). Each family received adapted and individualized counseling and was assigned to three practitioners (one physical activity and health educator, one nutrition and dietetics specialist, and one psychologist and social worker). Each practitioner made an initial two-hour introductory visit to the home, followed by six sessions with the family over the next six months. The topics of the educational sessions included definitions and benefits of physical activity and sedentary behaviors, such as “What do we mean by structured sport?” “How to be active at home?; definition and implication of weight loss and dietary restrictions; “What is a healthy and balanced diet?”, “How to anticipate and prepare a meal?”, and psychological sessions referred to “How to communicate within the family sphere?”, “How to handle screen time at the family level?”; and definition and identification of emotions and senses and stress management. Primary outcomes were children's and parents' evolution in z-BMI and BMI over the studied period. Outcomes were measured in four measurement occasions: at baseline/inclusion in the study, after the 6-month home-based intervention, after the 6-month independence phase (1 year from the inclusion), and after 12 months of the independence phase (2 years from the inclusion). The first results have shown a positive effect on BMI z-score reduction in children with obesity in 6 months and the importance of the family's composition as a predictor of completing the intervention (12). The significant changes in z-BMI observed in the short period after the home-delivered intervention (but not in the long term) reflect the need for support from families and are being considered by the study team for future editions. Although targeting children and adolescents already living with obesity, projects like ProxOb contribute to the body of evidence for home-based setting interventions, which are scarce, particularly for preventive strategies (13).

The JumpaKids is another German, community-based, low-threshold, ongoing free-of-charge counseling center established in 2019 for school-aged children and adolescents with overweight/obesity and their families (14). It provides an interdisciplinary care system and operates in close collaboration with local schools, kindergartens, physicians, youth welfare offices, and other institutions to improve the quality of life and wellbeing of children and adolescents who live with obesity and to promote health equity (15). The pilot phase (implemented for 3.5 years) shall prove to encourage children, adolescents, and their parents in Regensburg to adopt a health-promoting lifestyle through measures to motivate and promote skills in the areas of exercise, health-promoting nutrition, consumer skills, and social-emotional training, stress management, and addiction prevention. After 3 years of its establishment, more than 230 children and their families have asked for advice on eating habits, sports, and psychosocial difficulties. One of the primary outcomes is weight development, which is measured by BMI standard deviation scores. Initial results have proven that a 0.25 BMI-SDS overall reduction has been achieved after 3 months (14).

The role of primary healthcare units is essential in identifying and providing early intervention for obesity-related conditions such as diabetes and cardiovascular diseases. However, the escalating burden of obesity on healthcare systems and societal wellbeing necessitates a comprehensive, cross-disciplinary approach that encompasses biomedicine, social sciences, and humanities. This approach is crucial for identifying the underlying causes of obesity and designing effective interventions. To address these challenges, the PAS GRAS project, funded by the EU Horizon Europe Research and Innovation Action program (grant no. 101080329) and led by the University of Coimbra (Portugal), running until 2028, focuses on children (3–9 years), adolescents (10–18 years) and young adults (19–25 years) with overweight and obesity (16). The project aims to reverse obesity and prevent comorbidities later in life. By examining lifestyle factors (diet, sedentary behaviors, and physical activity), mental health, social/family influences, and environmental factors, the project seeks to understand how these elements interact with individual psychological, genetic, and metabolic characteristics. The project's highly qualified transdisciplinary team will integrate these parameters to develop personalized risk assessments for obesity and its complications. This will facilitate the co-production of innovative prevention and treatment strategies by integrating non-pharmacological lifestyle modifiactions with rational pharmacological targeting metabolic and neuroendocrine systems. Ultimately, the PAS GRAS project will address critical gaps in obesity diagnosis, prognosis, early interventions, obesity awareness, and food/nutrition literacy and provide a blueprint for a healthier population in Portugal and across the European Union (16). In this sense, this project will contribute to generating joint programs with healthcare centers, schools, health and sports clubs, municipalities, and involvement with public authorities, aiming at empowering everyone with the best possible strategy for preventing or reversing obesity across their lifespan. Recently, results from a study arm were presented in children aged 6–7 years (17). The team used visual methods like questions documented in drawings and game-based in-depth interviews using a memory game composed of wooden pieces with food subjects—open/closed fruits, vegetables, and a miscellaneous collection of food items, including processed food. First results highlighted the value of participatory, child-centered approaches in characterizing competences related to food to contribute to mapping environmental determinants of health from the children's perspectives.

This multi-country project addresses childhood and youth obesity through integrated research and intervention at all ecological levels, from individual to policy.

### Organizational level

At an organizational level, interventions target organizations and social institutions with rules and regulations, making schools a preferred setting to deliver preventive obesity interventions, as recently reviewed (13). School-based initiatives were presented as privileged areas for implementing prevention actions.

In Sweden, the STOPP project (18) aimed to assess the efficacy of a school-based intervention program to reduce rates of overweight in 6–10-year-old children over four school years between August 2001 and June 2005. The organizational strategies of the STOPP project focused mainly on changing the school environment so that it could be integrated as part of the ordinary school curriculum and possibly maintained within the ordinary school budget. Ten primary schools participated in the Stockholm county area (5 as control and five as intervention schools), including 3,135 children. The intervention proposed changing the foods offered at lunch and afternoon snacks within the school system to reduce unhealthy eating. More than 90% of the participating children ate lunch at school (offered free within the Swedish school system). Vegetables were offered as the first dish, white bread was substituted with a whole-grain version, and sugar content was reduced by eliminating fruit juices, soft drinks, and desserts, while fruit yogurt was replaced by plain yogurt. Additionally, skimmed milk (0.5% fat) was used instead of the regular versions, and low-fat butter, cheese, and yogurt were provided. Another aim was to increase physical activity and reduce sedentary behavior during school time by integrating 30 min of daily physical activity into the regular school curriculum by class teachers. Additionally, children were not allowed to bring devices to school and after-school care centers, increasing sedentary behavior (e.g., hand-held computer games), and the maximum time spent playing computer games at the after-school care centers was restricted to 30 min per child per day. All control schools continued their normal curriculum. An awareness intervention was done by sending newsletters to parents, and school staff off the intervention schools and school nurses received education in obesity-related problems. Outcome measurements were BMI-SDS, changes in physical activity, and eating habits at home. Pre and post-tests of weight and height were assessed over 3 years; the range between the baseline measurements varied between October 2001 and August 2004 and between May 2002 and June 2005 for the last measurements. Eating habits at home were assessed in the third and fourth grades at the end of the study using a questionnaire. For physical activity measurements, accelerometers were used, varying the range between the first and last measurements between April 2002 and June 2005. Over 4 years, a significant reduction of overweight and obesity by 3.2% in intervention schools was achieved (vs. a + 2.8% in control schools) (18). While physical activity did not differ between intervention and control schools, eating habits at home were healthier among families with children in intervention schools. Investigators recognized as a limitation that almost 1/3 of the children had participated in the project only for one year, and no control over summer holidays was implemented, which could affect the long-term results of the intervention, particularly in SDS-BMI evolution. In Austria, the EDDY (Effects of sports and diet training to prevent obesity and secondary diseases and to influence young children's lifestyle) project in Vienna (19), developed from 2022–2024 with support from the Federal Ministry of Education, Science and Research, was another example of preventive intervention at an organizational level. It aimed to tackle childhood obesity by implementing 10 h per semester of nutritional and physical activity education each (physical activity in addition to the two weekly hours that they receive by curricula) in the school setting. The project targeted children from 7–10 years old and included a nutritional intervention focused on age-appropriate nutrition education (through theoretical lessons) and its practical application in everyday life, including practical activities such as cooking and food tastings. Teachers were involved in the planning process, and newsletters were sent to parents to reinforce what had been taught regarding food, nutrition, and physical activity and to integrate healthy eating habits into family life. The physical activity intervention combined practical sports experience with theoretical education, including strength and endurance training. Outcomes were the evolution of anthropometric (SDS-BMI) and body composition (body fat, fat-free mass) parameters, subjective quality of life indicators, and motor skills using the German Motor Skills Test (DMT). The latter was measured on four occasions: at baseline (the start of the school year; September 2022), at the end of the school year (June 2023), at the start of the next school year (September 2023) and at the end of that school year (June 2024). Although the project was ongoing at the time of the symposium, during this report's elaboration, the first results were already available and therefore included. After 2 years, SDS-BMI in the intervention group remained stable, and total body fat percentage showed a minimal, non-significant reduction (−0.9%). Moreover, the intervention group showed increased health-related quality of life factors and motor skills compared to control group. These findings have been submitted for publication (manuscript under review). The EDDY project included different organizational strategies, such as improving weekly time dedicated to being in movement in school, promoting teachers' engagement through training to act as health promoters, and distributing communication materials throughout the school. However, one limitation mentioned is that there was no control over the summer period regarding eating and moving behaviors, which could affect the long-term intervention.

Another initiative presented during the symposium was the Health and Academic Performance with Happy Children (HAPHC) project in Graz, Austria, aiming to promote public health by increasing physical activity at an early age within the school setting (20). This ongoing project aims to reach∼3.000 healthy children of both sexes, aged 7–9 years (in grades 2–3), from 12 primary schools in Austria, Slovenia, and Belgium for 3 years. As physical activity shows to diminish with age and not every child has the opportunity or access to an adequate setting to practice it, the researchers attempt to integrate physical education in the school regular curriculum through the PAAC (Physical Activity Across the Curriculum) approach, based on the experience and findings of the Health-Oriented Pedagogical Project (HOPP) in Norway (21), which started in 2015 following children born in 2008 (1st grade in 2015) for 7 years. The HOPP consisted of adding 45 min of activity a day by replacing ordinary desk learning with physical tasks and the 90 min weekly physical education lessons children get in Norway according to the curriculum (21). All teachers of HAPHC intervention schools will receive training from experts from Norway and materials/teaching equipment that will allow them to adapt the pedagogy to their own culture and curriculum on different grades to integrate a daily physical activity unit of 45 min over 3 years across the curriculum. Children from schools acting as control groups will not receive active learning but will be taught according to the usual didactics. The following primary outcomes will be assessed: physical activity level and fitness, changes in anthropometric variables (BMI) and health-related physiological factors (such as blood lipids), academic achievement (through national tests for English, mathematics, and native languages), psycho-social aspects and wellbeing (using quality of life questionnaires, mapping of emotions and body image). The outcomes are intended to be measured at baseline, before the intervention starts, at the end of the first school year, and after the second and third years of implementation in all schools. Although primary intervention results are not available yet, recently, results from a secondary analysis exploring the association between subcutaneous adipose tissue and urine metabolites were presented as a thesis. Preliminary results from Austrian participating schools presented during the symposium and not published yet showed that all health and fitness variables consistently differed between the schools, indicating that physical fitness and related physical health largely depend on the children's socio-economic background. A reflection raised the socio-political question of whether providing social/financial resources can offer equal opportunities to all children growing up in Austria. This experience proposes a low-cost intervention, as school facilities and teachers as resources were already available; however, encouraging teachers to complete the intervention daily was challenging (21).

### Community level

While no intervention presented at the symposium was designed exclusively for the community level, some initiatives—such as JumpaKids and PAS GRAS—incorporate community elements through collaboration with local institutions, physicians and youth welfare offices to contribute to foster supportive environments.

### Policy level

Political commitment has been suggested as an important factor to fight childhood obesity in the long term (8). At a policy level, interventions aim to change policy or the environment in which the child lives. By operating upstream, they encourage context changes that decrease obstacles to individual behavior change, helping reform the obesogenic environment contributing to weight gain. The Symposium mentioned experiences from Germany, Portugal, and Denmark. Germany's way of advocating for structural condition-oriented obesity prevention, as proposed by the WHO, was present. Indicators on food taxation and regulation of food marketing in Germany have recently been rated very low to low (22). They are pursuing priority actions, such as developing a national policy to regulate food marketing in children and implementing a tax on sugary drinks. The German Obesity Society (DAG) and the German Association of Childhood Obesity (AGA) are working on this, focusing on enforcing comprehensive protection for children from harmful food advertising. In 2023, Germany's Federal Ministry of Food and Agriculture (BMEL) published the Children's Food Advertising Act, in which advertising to which children are exposed would be limited to products meeting the criteria of the 2023 edition of the Nutrient Profile Model of the WHO Regional Office for Europe. According to a recent report, across 22 product categories covered by the current draft law, the median share of products permitted for marketing to children stands at 55% (23). Considering the substantial share of products permitted for marketing to children across most product categories and the fact that the latest adaptations in the 2023 Children's Food Advertising Act have not convinced the critics within the German Government, an impact on the law's public health effect is still expected to be limited.

Additionally, in 2015, the German Government agreed on a National Strategy for the Reduction of Sugar, Fat, and Salt in Processed Foods, including a 15% reduction in the average sugar content of soft drinks in Germany between 2015 and 2025. In 2022, the elected German Government announced the implementation of a tax on sugar-sweetened beverages by the end of 2023 if voluntary measures by the industry were insufficient. A scientific publication initiated by researchers from Ludwig-Maximilians-Universität München (LMU Munich) reported that, until 2021, only a 2% reduction in the mean sales-weighted sugar content of soft drinks sold in Germany was achieved. Moreover, sugar sales from soft drinks in Germany decreased by 4% (22.4–21.6 g/capita/day) between 2015 and 2021; however, these rates remain high from a public health perspective (24). These results align with current evaluations by the Federal Research Institute for Nutrition (FoodMax Rubner-Institute), which, following a mandate by BMEL, reported on the sugar content of soft drinks on the German market in 2018 and 2020. Overall, the approach pursued by the German Government for voluntary sugar reduction in soft drinks is making little progress and has not fully achieved its stated targets (24).

On the same side, to give context to the PAS GRAS project initiative (previously mentioned), the strategies Portugal has taken to address the obesity pandemic were presented, as alarming facts projected an increase in childhood obesity rates of 3.5% per year between 2020 and 2035. Portugal has implemented policy changes to promote healthy lifestyles (25), including regulating the marketing of unhealthy foods and reducing salt content, particularly in products like bread, as part of a national food reformulation plan with the participation of the food industry and retailer sectors. In 2017, a soft drinks tax was created (26). After a year of implementation, several beverages' sugar contents were reduced, leading to an 11% reduction in total energy intake through sweetened beverages consumption by the Portuguese population (26). From January 2019, the tax was redesigned to maximize sugar reduction and reformulation among producers (an additional 15% reduction of the energy amount on a progressive scheme). The co-regulation agreement with the food industry implemented in 2018 defined food reformulation targets: the reduction of 16% for salt (plus reducing salt in bread by 30%), 20% for sugar and a limit of 2 g trans-fatty acids per 100 g of fat in margarine and shortening (and limiting 1 g per 100 g of fat in pastry) by 2021 (27). Overall, during the last decade in Portugal, a shift in actions more focused on the obesogenic environment has been implemented, contributing to making good progress in implementing suggested policies to tackle the obesity problem; however, challenges in implementation are still present (25).

Denmark has led a unique strategy to tackle the childhood obesity problem, which extended health registers, and strong collaboration between health authorities, the private sector, and research communities can summarize. In 1999, the Government published a 10-year action plan for public health, considering obesity as a significant health issue for the first time in government plans. The plan's central part concerns childhood obesity prevention and weight reduction to reduce associated complications (28). It also includes adults. One of the significant contributions to pediatric obesity in Denmark has been The Copenhagen School Health Records Register, operating from 1942 onwards and covering information on 372,636 children from 1936–2005. It is an electronic record of health examination information, including serial weight, height measurements, and birth weight. Personal unique identification numbers make linkages possible to national registers and population studies. Every single schoolchild is included in the register. Universities and University Hospitals' contribution to pediatric obesity was also highlighted; however, advocating for efforts more focused on preventing and managing pediatric obesity, the Government, the private sector, and the Novo Nordisk Foundation have recently established a “Centre for Childhood Health.” (29) Towards 2026, they plan to work on three major points: (a) generate more research on causes of excessive weight, (b) work with local councils, civil society, citizens, and retailers to build up ideal daily healthy municipalities, (c) remove prejudices around body size and weight stigma. For example, under the initiative “Only Water,” the Centre for Childhood Health surveyed 3,000 children and young people aged 3–20 to map their drinking habits and whether everyone can access water stations in schools, clubs, and extracurricular activities (29). The experience of extended health registers and the strong collaboration between health authorities, the private sector, and research communities summarizes the unique strategies of Denmark to tackle the childhood obesity problem, with a plan that includes national goals, regulatory measures, and innovative programs. The national plan operates at all levels of the EST, integrating individual behavior change, family and school involvement, organizational standards, community initiatives, and national policy to create a comprehensive, multi-level response to obesity.

Sweden also has a national initiative called “Ending Childhood Obesity,” a strategic project for a system transformation toward obesity prevention in children aged 0–6 (30). It aims to end childhood obesity before starting school by 2030; however, long-term results are being threatened by a lack of finances. The Swedish innovation program SWElife started a project divided into five work packages-WP (from individual initiatives to incentives and business models) to increase the small proportion of health costs that go to prevention. In Sweden, three percent of health care costs go to prevention. One WP focused on individual actions and performed a survey to find, to date, ongoing, evidence-based initiatives to work with children living with obesity and overweight. A systematic review of the literature was also performed (31). Primary results have highlighted that individual programs working with children with obesity and overweight are more effective if societal and political actions support them. The use of established models and evaluation implementation to improve future programs was highlighted. Consequently, another WP included a Grand Challenge set up in 2021. This national competition used a Hackathon to find new initiatives to generate system-changing solutions that could orient toward the vision of Zero Childhood Obesity at school start by 2030. The top projects, such as digital twins and apps supporting families, were selected and presented to the market, but further evaluation was stopped recently as the project needed further funding. The WP Measurements, Follow-up, and Data processing were in charge of producing terminology and data analysis for prevention efforts against childhood obesity to facilitate digital working methods. Suitable databases were identified to find data for further evaluation, and the problem with personal integrity was suggested to be solved by using self-reported data. The WP Incentives, Reimbursement, and Business models searched for ways to make preventive healthcare a financially sustainable business. In this sense, finding new suitable business models was challenging, although several were evaluated, such as support from insurance companies and governmental support (Appendix, Table 2). A literature search evaluated models with a beneficial health economy to create a better understanding and basis for developing, testing, and implementing incentives, reimbursement, and business models for preventive interventions for the target group of children 0–6 years. They concluded that there is a need for more health economic studies on childhood obesity (32). Finally, systems approaches were recommended, as suggested by the guidance from Public Health England. However, to achieve results long-term, financial support is needed. Sweden's initiative addresses childhood obesity at all levels of the EST by integrating individual and family support, organizational and community engagement, and national policy transformation. It exemplifies a comprehensive, systems-oriented approach to prevention, where the public sector, non-governmental organizations and the private sector unite aiming for lasting change in Sweden's youngest generation.

### Practical insights

Presenters at the symposium highlighted that obesity prevention and management should focus beyond the individual or school domain, including actions across multiple settings. Even individual programs get more effective if societal or political actions support them. Cooperation and joint responsibility from all actors involved were stated as important; everyone participating should “own” the initiative for sustainability.

A clear takeaway was the importance of tailoring interventions to local contexts, considering cultural norms, socioeconomic challenges, and available community resources. Early intervention emerged as critical, with the most significant potential impact seen when healthy behaviors are promoted from infancy and reinforced throughout childhood. Presentations also advocated for embedding health promotion within existing institutional frameworks (such as school curricula and primary care practices) to ensure both scalable and sustainable interventions, considering teachers' motivation and reachability to parents/caregivers, as factors like migration background, level of education, language comprehension, or religion may act as barriers. Using incentives (economic or material), remainders, and feedback support was suggested to increase compliance. Although the ideal time frame for preventive strategies to be successful was shown to be variable, most of the initiatives included a duration beyond one year, using a control school, and including a home element.

Furthermore, monitoring and evaluating initiatives using standardized outcomes—for example, BMI z-scores and body composition measures—was emphasized to facilitate comparison and ongoing improvement. Ultimately, a shift toward a whole-systems approach—where intervention efforts extend beyond individuals to influence social, environmental, and policy determinants—was recommended to achieve equity and lasting change in childhood obesity rates.

## Conclusion

Despite acknowledging that prevention is the way to go to tackle childhood obesity and the support for multicomponent interventions, there are still substantial shortcomings observed from European experiences. Overall, family and school-based interventions are considered best practices.

Coordinated actions across all levels of the EST are necessary to gather successful experiences in childhood obesity prevention. When actions at a policy level were presented, a more comprehensive approach to the problem was observed. In this sense, dialogues in politics, therefore, should be permanent in terms of obesity. In terms of taxation regimens, based on the Portuguese experience, these should be flexible enough to be reviewed and changed according to available evidence and in favor of public health interests. While such presented interventions seem promising, it is acknowledged by researchers that financial support for their long-term success is crucial. Lastly, it was evident from the presentations that focused on treatment strategies during the symposium that obesity during childhood is a problem, and particular strategies are needed. In summary, while most interventions presented focused on the individual, interpersonal, and organizational levels—such as family-based counseling, school programs, and healthcare initiatives—fewer targeted the broader community or policy environment. This highlights an opportunity for future efforts to develop interventions that operate at all levels of the ecological system to achieve sustainable and equitable achievements in childhood obesity.

## Data Availability

The original contributions presented in the study are included in the article/Supplementary Material, further inquiries can be directed to the corresponding author.

## Appendix

**Table 1:** Classification of symposium interventions for childhood obesity prevention and treatment using the ecological systems theory framework.

Settings	Name	Type	Country	Interventions	Characteristics	Outcomes	Ref.
Pediatric practice	STARKIDS	Treatment	Germany. Baden-Wuerttemberg	Step 1: face-to-face training and an e-health online platform for 1 year. Step 2: if not successful BMI reduction, one to two counseling sessions during 6 months.	Modules on topics: “Eating and Drinking,” “Activity and media,” “Family life,” “STARKIDS Keep on,” and “STARKIDS Joker” (optional), including educative/interactive texts, animated films, reflection tools and/or serious games.	- z-BMI development and positive change in children's and adolescents' quality of life.	(10)
Home	ProxOb	Treatment	France	- 6-month multidisciplinary, home-based education intervention; - 18-month maintenance phase with regular phone support.	- A 40 h course on obesity was delivered to trainers. - Adapted and individualized counseling by 3 practitioners: physical activity and health educator, nutrition and dietetics specialist, and psychologist and/or social worker. - 2 h introductory visit + six visits sessions by each practitioner during 6 months.	- Children's and parents' evolution in z-BMI and BMI over the studied period.	(12)
Community	JumpaKids	Treatment	Germany. Regensburg	Individual counseling, group programs, physical activity promotion, nutritional counseling, and psychosocial support.	Operates closely collaboration with local schools, kindergartens, physicians, youth welfare offices, and other institutions.	- z-BMI development.	(14)
Clinical, community, schools, and family.	PAS GRAS	Prevention (and elements for treatment)	Portugal, Italy, Germany, the United Kingdom, Poland, Sweden, and Spain.	- Integrated analysis of lifestyle, mental health, family, socioeconomic, environmental, genetic, and metabolic factors. - Development of personalized risk assessments for obesity and related complications. - Study of cellular and molecular mechanisms, especially the protective effects of the Mediterranean diet and physical activity. - Co-production of creative and interactive health literacy tools and interventions. - International campaigns to increase health literacy and raise awareness about obesity risks.	- Implement personalized, risk-adapted interventions (dietary, physical activity, pharmacological). - Launch of an international campaign to increase health literacy and societal awareness about obesity.	- Improved understanding of the multifactorial causes of obesity, personalized obesity risk assessment, increased health literacy, and societal obesity awareness.	(16)
School	STOPP	Prevention	Sweden. Stockholm	- School lunch was modified to increase fiber content and reduce saturated fat and sugary product intake. - Physical activity increases by 30 minutes during school time, and sedentary behavior is restricted during and after school care time. - Newsletters were sent to parents and school staff.	- Low-fat dairy products and whole-grain bread were promoted in school meals, and all sweets and sweetened drinks were eliminated in intervention schools. - Parents were encouraged to eliminate sweat products and beverages during festivities and excursions. - Dimensions assessed: eating behavior, eating attitude, and eating disorders.	- Changes in SDS-BMI, physical activity during school time, and changes in eating habits at home.	(18)
School	EDDY	Prevention	Austria. Vienna	- Nutritional intervention (through theoretical lessons) and practical application in everyday life. - Teachers were involved. - Newsletters were sent to parents. - Physical activity intervention	- Content focusing on food with high nutrient content, relevance of timing in eating, skills to prepare and select healthier food. - Choices (e.g., recipes for easy, healthy snacks) and identification of suitable portion sizes. - Physical activity intervention combining strength and endurance training.	- Evolution of anthropometric and body composition parameters, subjective quality of life indicators, and motor skills (German Motor Skills Test -DMT).	(19)
School	HAPHC	Prevention	Austria, Slovenia, and Belgium	-Daily physical activity unit of 45 min over 3 years across the curriculum. -Teachers receive training.	Uses the PAAC (Physical Activity Across the Curriculum) approach.	- Changes in anthropometric variables, physical activity and fitness, health-related physiological factors, academic achievement, psycho-social aspects, and wellbeing.	(20)
Policy		Prevention	Germany	- National policy to regulate food marketing in children. - Tax on sugary drinks.	- Advertising is limited to products meeting the criteria of the 2023 edition of the Nutrient Profile Model (WHO). - Target: 15% reduction in sugar content of soft drinks between 2015 and 2025.	- Reduction in beverages' sugar content and a reduction in means sales of sugary drinks.	(23,24)
Policy		Prevention	Portugal	- National food reformulation plan. - Tax on sugary drinks.	-Reduction targets for salt, sugar, and trans-fatty acids in foods. - Taxes implemented according to sugar content: drinks with sugar contents below 80 g/L of the final product, charged at €8.22 per 100 L; above 80 g/L of the final product, charged at €16.46 per 100L.	- Reduction in sugar, salt, and trans-fatty acids in foods. - Reduction in sugar content of beverages.	(25,26,27)
Policy	National Action Plan Against Obesity	Prevention/Treatment	Denmark	-Evidence-based recommendations for diet and physical activity. -School-based programs and standards. -Restrictions on unhealthy food marketing. -Community and workplace health promotion.	Also, a pilot and innovative treatment program for those already living with severe obesity.	- Reduction in obesity and overweight prevalence. - Improved health literacy and healthy habits. Decreased social inequality in health. Delayed onset of obesity-related diseases.	(28)
Policy	Ending Childhood Obesity	Prevention	Sweden	-System transformation through multi-sector collaboration. -Five work packages (WP). -Data-driven approach for monitoring and evaluation. -Incentivizes preventive actions through new business and reimbursement models. -National competitions to generate innovative solutions.	WP: national coordination, implementation, best practices, data management, and incentives/business models.	- Reduction in overweight and obesity among children under 6. - Improved health equity. - Increased investment in preventive health. - Enhanced data infrastructure and knowledge sharing.	(30)

**Table 2:** Summary of incentives, reimbursement, business models and health economic models for preventive interventions against childhood obesity.

Bussisness model type	Description	Economic evaluation	Challenges
Governmental funding	Public budgets allocated for childhood obesity prevention at regional and municipal levels.	Seen as essential for sustainable prevention; supports long-term cost-effectiveness.	Sustainability depends on political will; needs alignment across sectors.
Reimbursement models	Budget, capitation, fee-for-service, and result-based payments mainly designed for treatment.	Lack of evidence for incentivizing prevention outcomes; treatment-focused.	May cause administrative burden; needs integration with other governance tools. Its importance should not be exaggerated.
Public-Private Partnerships	Collaboration between government, NGOs, and private businesses for prevention efforts.	Potential for cost-sharing and wider reach; direct economic data limited.	Coordination complexity, and risk of conflicting priorities; private sector often lacks prevention focus.
Local collaborative initiatives	Community-based multi-actor groups coordinating prevention activities (e.g., Kungsbacka-Sweeden).	Positive local effects; informal success with limited formal funding.	Often depend on personal commitment; needs funded coordination for scaling and sustainability.
Private sector business models	Commercial actors focus on value creation; prevention rarely a core business aim.	Short-term profit motives conflict with prevention goals.	Misaligned incentives make prevention incorporation challenging; need regulatory and collaborative frameworks.
Social outcome contracts	Risk-sharing agreement linking payments to measurable social/health outcomes.	Early pilots (e.g., Norrköping-Sweeden) show promise for scalable, cost-effective solutions.	Complex coordination; novel approach with limited testing in childhood obesity prevention.
Market regulations and policies	Laws and rules like sugar taxes, advertising restrictions targeting childhood obesity.	International evidence (e.g., Chile, UK) supports effect on changing consumer behavior and economic gains.	Politically sensitive; requires ethical and economic support.
Behavioral Incentives (Nudges, Taxes)	Soft policy tools influencing behavior towards prevention (e.g., nudges, taxes, subsidies).	Some evidence of public health and economic benefits.	Limited effectiveness alone; ethical debates exist; political acceptance varies.

This summary was based on the report of the Swelife initiative, Prevention of childhood obesity, with the vision of Zero obesity at the start of school by 2030. Sweden (32).
